# Supplementary material for: Content-rich biological network constructed by mining PubMed abstracts
Source: BMC Bioinformatics. 2004 Oct 8;5:147. doi: 10.1186/1471-2105-5-147 (PMC528731; doi:10.1186/1471-2105-5-147)
Supplement: Additional File 2 — The original results of the above study (non-essential files are deleted to keep the file size under the limit set by BMC bioinformatics). [file 1471-2105-5-147-S2.bz2 › chilibotAdditionalFile2/dip50/25ID8999548E88/html/CASP1_IL1B.html]

 


 **CASP1** and **IL1B** 
  
Found 435 abstracts in PubMed, retrieved 50.  
 

 What does Google say? 
 PDF only 
| .edu only 

---

**Interactive relationship** (e.g. stimulation, inhibition, etc)

**Stimulatory relationship**- After immune or stress stimulation, ICE  [ **CASP1** ]  deficient mice were shown to have a hyperactive HPA axis and to able to produce immunoreactive IL 1 beta  [ **IL1B** ] .  Ref: 11268342 Ann N Y Acad Sci, 2000
- Furthermore, treatment with the IL 1 beta  [ **IL1B** ]  converting enzyme  [ **CASP1** ]  ICE  [ **CASP1** ]  specific reversible inhibitor YVAD CHO resulted in a sharp dose and time dependent inhibition of IL 1 beta  [ **IL1B** ]  secretion in the serum.  Ref: 11399517 Eur Cytokine Netw< MedlineTA>Eur Cytokine Netw, 2000
**Neutral relationship**- IL1 beta  [ **IL1B** ]  mRNA expression was parallel to ICE  [ **CASP1** ]  mRNA expression.  Ref: 10923469 Zhonghua Yi Xue Za Zhi, 1998
- Interleukin 1 IL 1 beta  [ **IL1B** ]  IL 1beta converting enzyme ICE  [ **CASP1** ]  is a cysteine protease caspase 1  [ **CASP1** ]  that processes inactive 33 kDa pro IL 1beta to the active 17 kDa inflammatory cytokine.  Ref: 11754996 Neurobiol Aging< MedlineTA>Neurobiol Aging, 1998
- caspase 1  [ **CASP1** ]  is the intracellular protease that cleaves the precursors of IL 1 beta  [ **IL1B** ]  and IL 18 into active cytokines.  Ref: 11606779 Proc Natl Acad Sci U S A, 2001
- Amebic lysates have also been shown to activate the precursor to interleukin 1 beta  [ **IL1B** ]  proIL 1beta, mimicking the action of caspase 1  [ **CASP1** ] .  Ref: 12595442 Infect Immun, 2003
- Mature IL 1 beta  [ **IL1B** ]  derives from a precursor, the pro IL 1 beta  [ **IL1B** ] , devoid of any conventional signal sequence that is mainly processed by ICE  [ **CASP1** ] .  Ref: 11268342 Ann N Y Acad Sci, 2000
- This indicates that the greater reactivity of the HPA axis could result from a higher sensitivity to non ICE  [ **CASP1** ]  matured IL 1 beta  [ **IL1B** ] , as suggested by a higher basal transcription of hypothalamic IL 1 receptor type I IL 1 RI in ICE  [ **CASP1** ]  deficient mice.  Ref: 11268342 Ann N Y Acad Sci, 2000

**Non-interactive relationship** (e.g. studied together, co-existance, homology, etc.)

- To study the pathophysiological roles of overexpressed caspase 1  [ **CASP1** ]   **CASP1** , originally designated as IL 1 beta  [ **IL1B** ]  converting enzyme  [ **CASP1** ] , we generated transgenic mice in which human  **CASP1**  is overexpressed in their keratinocytes.  Ref: 10878376 J Immunol, 2000
- Correlation of interleukin 1 beta  [ **IL1B** ]  converting enzyme  [ **CASP1** ]  ICE  [ **CASP1** ]  gene expression with gut epithelial cell apoptosis in septic mice < ArticleTitle>.  Ref: 10923469 Zhonghua Yi Xue Za Zhi, 1998
- Co expression of PYPAF7 and ASC results in the synergistic activation of caspase 1  [ **CASP1** ]  and a corresponding increase in secretion of interleukin 1 beta  [ **IL1B** ] .  Ref: 12019269 J Biol Chem, 2002
- During the postnatal period of development, when facial motoneurons are highly vulnerable to axotomy, we did not observe any significant effect of the interleukin 1 beta  [ **IL1B** ]  converting enzyme  [ **CASP1** ]  deletion on the percentage of cell death in the lesioned nuclei.  Ref: 10869851 Neuroscience, 2000
- The mRNA expression of IL 18 and IL 1 beta  [ **IL1B** ]  converting enzyme  [ **CASP1** ]  was significantly reduced in unstimulated monocytes from patients with AD P < ;.  Ref: 11590389 J Allergy Clin Immunol, 2001
- Shigella flexneri infection of human macrophages is followed by rapid bacterial escape into the cytosol and secretion of IpaB, which activates caspase 1  [ **CASP1** ]  to mediate cell death and release of mature interleukin IL 1 beta  [ **IL1B** ] .  Ref: 12065527 Infect Immun, 2002
- Using primary cultured murine peritoneal macrophages, we demonstrate that P2X7 receptor activation causes release of IL 1 beta  [ **IL1B** ]  and IL 1 alpha via a common pathway, dependent upon the release of Ca 2 from endoplasmic reticulum stores and caspase 1  [ **CASP1** ]  activity.  Ref: 12626557 J Immunol, 2003
- The LPS induced effects were abrogated by inhibition of caspase 1  [ **CASP1** ] , suggesting that IL 1 beta  [ **IL1B** ]  may mediate the effects of LPS.  Ref: 12609991 J Biol Chem, 2003
- Effect of TNF alpha on the induction of apoptosis in murine macrophages role of interleukin 1 beta  [ **IL1B** ]  converting enzyme  [ **CASP1** ] .  Ref: 12622883 Int J Immunopathol Pharmacol< MedlineTA>Int J Immunopathol Pharmacol, 2003
- Changes in caspase 3 and caspase 1  [ **CASP1** ] , interleukin 1 beta  [ **IL1B** ]  IL 1 beta  [ **IL1B** ] , and collagen I, III, and IV proteins and mRNA were detected by Western blotting and Northern blotting, respectively.  Ref: 12753292 Kidney Int, 2003
- RESULTS Expression of TNF alpha, IL 1 beta  [ **IL1B** ] , caspase 1  [ **CASP1** ]  and NOS2 was found in the cytoplasm and sarcolemma of dystrophic skeletal muscle fibres.  Ref: 12673888 Clin Exp Rheumatol< MedlineTA>Clin Exp Rheumatol, 2003
- OBJECTIVE To investigate the effects of Arg Gly Asp Ser RGDS tetrapeptide on the expressions of apoptosis related genes interleukin 1 beta  [ **IL1B** ]  converting enzyme  [ **CASP1** ]  ICE  [ **CASP1** ]  and bcl 2 in human glomerular mesangial cells, so as to provide experimental evidence for regulating proliferation and apoptosis of mesangial cells with RGD containing polypeptide.  Ref: 10923408 Zhonghua Yi Xue Za Zhi, 1998
- The number of epithelial cell apoptosis was correlated excellently to the level of ICE  [ **CASP1** ] , IL1 beta  [ **IL1B** ]  mRNA expression.  Ref: 10923469 Zhonghua Yi Xue Za Zhi, 1998
- It lacks a signal peptide in common with other known IL 1B  [ **IL1B** ]  genes, and lacks a clear ICE  [ **CASP1** ]  caspase cut site in common with other nonmammalian IL 1B  [ **IL1B** ]  genes sequenced to date.  Ref: 10803846 Immunogenetics, 2000
- We have also assessed the immunoreactivity of interleukin 1 beta  [ **IL1B** ]  converting enzyme  [ **CASP1** ] , caspase 1  [ **CASP1** ] , a key enzyme in the execution of apoptosis, and of P cadherin, which has been suggested as a key adhesion receptor in segregating proliferating keratinocytes.  Ref: 11407986 J Invest Dermatol, 2001
- Entamoeba histolytica cysteine proteinases with interleukin 1 beta  [ **IL1B** ]  converting enzyme  [ **CASP1** ]  ICE  [ **CASP1** ]  activity cause intestinal inflammation and tissue damage in amoebiasis.  Ref: 10931347 Mol Microbiol, 2000
- TNF alpha increased the messenger ribonucleic acid levels of ICE  [ **CASP1** ] , TNF alpha, IL 1 beta  [ **IL1B** ] , bcl 2, and NF kappa B in preadipocytes and adipocytes P <.  Ref: 11397893 J Clin Endocrinol Metab, 2001
- PURPOSE To detect cell specific apoptosis factors, Fas and Fas ligand, and the common intracellular apoptosis modulators, interleukin 1 beta  [ **IL1B** ]  converting enzyme  [ **CASP1** ]  ICE  [ **CASP1** ]  like protease caspase 1  [ **CASP1** ] , Bcl 2, Bcl xL and Bax in lens epithelial cells LEC of human cataracts.  Ref: 11803481 Curr Eye Res, 2001
- IL1 beta  [ **IL1B** ] , ICE  [ **CASP1** ]  gene expression was detected quantitatively by RT PCR.  Ref: 10923469 Zhonghua Yi Xue Za Zhi, 1998
- Of the 4 cDNA fragments, 2 A1 and D1 did not show sequence homology with the existing genes in the GeneBank, one A2 was 89% homologous with interleukin 1 beta  [ **IL1B** ]  converting enzyme  [ **CASP1** ]  ICE  [ **CASP1** ]  gene, and the other D2 was identical to mouse musculus 45s pre rRNA MM45srRNA gene.  Ref: 11783011 Zhonghua Zhong Liu Za Zhi, 2001
- Using RT multiplex PCR, the expression of the proapoptotic genes interleukin 1 beta  [ **IL1B** ]  IL 1 beta  [ **IL1B** ]  converting enzyme  [ **CASP1** ]  ICE  [ **CASP1** ]  and TNF alpha and the antiapoptotic genes bcl 2, nuclear factor kappa B NF kappa B, and NF kappa B inhibitory subunit, I kappa B, were examined.  Ref: 11397893 J Clin Endocrinol Metab, 2001
- During the early stages of hair follicle development, interleukin 1 beta  [ **IL1B** ]  converting enzyme  [ **CASP1** ]  immunoreactivity was present on all keratinocytes, but virtually disappeared from the proximal hair follicle epithelium later on.  Ref: 11407986 J Invest Dermatol, 2001
- OBJECTIVE To investigate the correlation of gut epithelial cell apoptosis with ICE  [ **CASP1** ] , IL1 beta  [ **IL1B** ]  gene expression in septic mice.  Ref: 10923469 Zhonghua Yi Xue Za Zhi, 1998
- We report that potassium leakage from cells leads to activation of the Ca2 independent phospholipase A2 iPLA2, and the latter plays a pivotal role in regulating the cleavage of pro IL 1 beta  [ **IL1B** ]  by the IL converting enzyme caspase 1  [ **CASP1** ]  in human monocytes.  Ref: 10799869 J Immunol, 2000
- They were first studied in mice deficient for the IL 1 beta  [ **IL1B** ]  converting enzyme  [ **CASP1** ]  ICE  [ **CASP1** ]  gene.  Ref: 11268342 Ann N Y Acad Sci, 2000
- The critical role of amoebic cysteine proteinases in human gut inflammation and tissue damage may be explained by our discovery that amoebic cysteine proteinases possess IL 1B  [ **IL1B** ]  converting enzyme ICE  [ **CASP1** ]  activity.  Ref: 10931347 Mol Microbiol, 2000
- Activation of the hypothalamic pituitary adrenal axis in IL 1 beta  [ **IL1B** ]  converting enzyme  [ **CASP1** ]  deficient mice.  Ref: 10810251 Neuroimmunomodulation, 2000
- As cytoplasmatic adaptor molecules of FAS, e.g. FLIP Fas associated death domain protein FADD like interleukin 1 beta  [ **IL1B** ]  converting enzyme  [ **CASP1** ]  FLICE caspase 8 inhibitory protein, also modulate TRAIL signals, we determined whether chelerythrin affected TRAIL mediated apoptosis.  Ref: 12877678 Br J HaematolBr J Haematol, 2000
- Among these caspases, several in vitro and in vivo reports indicate that the interleukin 1 beta  [ **IL1B** ]  converting enzyme  [ **CASP1** ]  or caspase 1  [ **CASP1** ]  may be involved in neurodegenerative processes.  Ref: 10869851 Neuroscience, 2000
- Although decreased snRNP protein could be explained by decreased mRNA steady state levels, another underlying mechanism might be suggested snRNP is one of the death substrates rapidly cleaved during apoptosis by interleukin 1 beta  [ **IL1B** ]  converting enzyme  [ **CASP1** ]  like ICE  [ **CASP1** ]  proteases, which was well documented by several groups.  Ref: 11197177 Electrophoresis, 2001
- Identification of interleukin 1 beta  [ **IL1B** ]  converting enzyme  [ **CASP1** ]  gene expression in human kidney < ArticleTitle>.  Ref: 10923536 Zhonghua Yi Xue Za Zhi, 1998
- Intriguingly, unlike the caspase 8 inhibitor cellular FADD like interleukin 1 beta  [ **IL1B** ]  converting enzyme  [ **CASP1** ]  inhibitory protein c FLIP, NDED suppressed TNF mediated apoptosis by inhibiting TNF induced caspase 8 enzymatic activity but not the processing of caspase 8.  Ref: 11346652 J Biol Chem, 2001
- Vertex is collaborating with Aventis Pharma AG formerly Hoechst Marion Roussel Inc in the development of pralnacasan, an interleukin IL 1b  [ **IL1B** ]  converting enzyme ICE  [ **CASP1** ]  inhibitor, for the potential treatment of inflammatory diseases 17024 8829 53094.  Ref: 12789619 Idrugs, 2003
- OBJECTIVE To investigate the effects of interleukin 1 beta  [ **IL1B** ]  converting enzyme  [ **CASP1** ]  ICE  [ **CASP1** ]  gene on the biologic characteristics of ovarian cancer cells.  Ref: 11263197 Zhonghua Fu Chan Ke Za Zhi, 1999
- METHODS Immunohistochemical staining of skeletal muscle biopsies 10 DM, 10 SSc, 10 PM, 10 SLE, 10 non inflammatory myopathies for tumour necrosis factor alpha TNF alpha, interleukin 1 beta  [ **IL1B** ]  IL 1 beta  [ **IL1B** ] , activated caspase 1  [ **CASP1** ] , pan macrophage marker CD68, inducible nitric oxide synthase NOS2 and nerve growth factor receptor NGFR.  Ref: 12673888 Clin Exp Rheumatol< MedlineTA>Clin Exp Rheumatol, 1999
- CONCLUSION Prominent expression of the proinflammatory factors TNF alpha, IL 1 beta  [ **IL1B** ]  and NOS2 and caspase 1  [ **CASP1** ]  is associated with muscle fibre damage, albeit when expressed to a low degree these factors may, like NGFR, contribute to muscle regeneration and healing.  Ref: 12673888 Clin Exp Rheumatol< MedlineTA>Clin Exp Rheumatol, 1999
- Both TNF alpha and IL 1 beta  [ **IL1B** ]  are important signals governing this process, but the potential regulatory role of IL 1 alpha processing by caspase 1  [ **CASP1** ]  is unknown.  Ref: 11238606 J Immunol, 2001
- Gene expression of mRNA for IL 1alpha, IL 1beta, IL 1 receptor antagonist IL 1ra, type 1 IL 1 receptor, type 2 IL 1 receptor, and IL 1 beta  [ **IL1B** ]  converting enzyme  [ **CASP1** ]  ICE  [ **CASP1** ] , were measured by a semi quantitative RT PCR technique.  Ref: 11137121 Vet Immunol Immunopathol, 2000
- IL 1 beta  [ **IL1B** ]  converting enzyme  [ **CASP1** ]  is a target for nitric oxide releasing aspirin new insights in the antiinflammatory mechanism of nitric oxide releasing nonsteroidal antiinflammatory drugs.  Ref: 11046058 J Immunol, 2000
- Bromoenol lactone dependent inhibition of IL 1 beta  [ **IL1B** ]  processing was not due to perturbation of the export machinery for pro IL 1 beta  [ **IL1B** ]  and IL 1 beta  [ **IL1B** ]  or to caspase 1  [ **CASP1** ]  suppression.  Ref: 10799869 J Immunol, 2000
- We sought to determine whether mice deficient in the proinflammatory caspase 1  [ **CASP1** ] , which cleaves precursors of IL 1 beta  [ **IL1B** ]  and IL 18, were protected against ischemic acute renal failure ARF.  Ref: 11342578 J Clin Invest, 2001
- Inhibition by SK& ; F 86002 and interleukin 1 beta  [ **IL1B** ]  converting enzyme  [ **CASP1** ]  inhibitor.  Ref: 11399517 Eur Cytokine Netw< MedlineTA>Eur Cytokine Netw, 2001
- No detectable levels of p53, Bax, Bcl 2 and the subunit p20 of interleukin 1 beta  [ **IL1B** ]  converting enzyme  [ **CASP1** ]  ICE  [ **CASP1** ]  were found by Western blot, but the active subunit p17 of 32 kDa putative cysteine protease CPP32 was detected at, and 9 h after ricin treatment.  Ref: 11324424 Acta Pharmacol Sin, 2000
- In vivo study of motoneuron death induced by nerve injury in mice deficient in the caspase 1  [ **CASP1** ]  interleukin 1 beta  [ **IL1B** ]  converting enzyme  [ **CASP1** ] .  Ref: 10869851 Neuroscience, 2000
- OBJECTIVE To confirm that Interleukin 1 beta  [ **IL1B** ]  converting enzyme  [ **CASP1** ]  ICE  [ **CASP1** ]  is a first identified apoptosis related gene in mammals.  Ref: 10923536 Zhonghua Yi Xue Za Zhi, 1998
- IL 1 beta  [ **IL1B** ]  converting enzyme  [ **CASP1** ]  ICE  [ **CASP1** ] .  Ref: 11606779 Proc Natl Acad Sci U S A, 2001
- The expression and release of IL 1 beta  [ **IL1B** ] , a postulated downstream effector of ICE  [ **CASP1** ]  mediated apoptosis, were also determined.  Ref: 11397893 J Clin Endocrinol Metab, 2001
- Dual effects of caspase 1  [ **CASP1** ] , interleukin 1 beta  [ **IL1B** ] , tumour necrosis factor alpha and nerve growth factor receptor in inflammatory myopathies.  Ref: 12673888 Clin Exp Rheumatol< MedlineTA>Clin Exp Rheumatol, 2001
- IL1 beta  [ **IL1B** ] , ICE  [ **CASP1** ]  mRNA expression in CLP mice was significantly higher than that in the sham operation group P <.  Ref: 10923469 Zhonghua Yi Xue Za Zhi, 1998
- In this study we demonstrate that caspase 1  [ **CASP1** ]  like proteases and interleukin 1 beta  [ **IL1B** ]  are important for death induced by various stimuli in cell lines, primary fibroblasts and primary sensory neurons.  Ref: 11232240 Apoptosis, 2000
- The terminal stages of cell death, including death of neurons, depend on the activation of caspases, specifically caspase 1  [ **CASP1** ]  interleukin 1 beta  [ **IL1B** ]  converting enzyme  [ **CASP1** ] .  Ref: 11544763 Ontogenez< MedlineTA>Ontogenez, 2000
- Cells on oxidized matrix demonstrated enhanced expression of Bax, increased fragmentation of PARP, and diminished apoptosis in the presence of the interleukin 1 beta  [ **IL1B** ]  converting enzyme  [ **CASP1** ]  inhibitor Ac Tyr Val Ala Asp aldehyde.  Ref: 11922616 Biochem Biophys Res Commun, 2002
- Dex inhibited TNFalpha induced messenger ribonucleic acid expression of ICE  [ **CASP1** ] , TNF alpha, and IL 1 beta  [ **IL1B** ]  P <.  Ref: 11397893 J Clin Endocrinol Metab, 2001
- Intradermal IL 1 beta  [ **IL1B** ]  50 ng caused a similar reduction in epidermal LC numbers in both WT and caspase 1  [ **CASP1** ]  deficient mice, indicating that, given an appropriate signal, caspase 1  [ **CASP1** ]  deficient epidermal LC are capable of migration.  Ref: 11238606 J Immunol, 2001
- In the lining like layers and deep stroma of SMLIT, many cells were double positive for active caspase 1  [ **CASP1** ]  and interleukin 1 beta  [ **IL1B** ]  IL 1beta.  Ref: 12111083 Rheumatol Int, 2002
- These results suggest that lemon pure essential oils may modulate the apoptosis through the activation of the interleukin 1 beta  [ **IL1B** ]  converting enzyme  [ **CASP1** ]  like caspases.  Ref: 12162947 Pharmacol Res, 2002
- In response to ATP pro IL 1 beta  [ **IL1B** ]  produced by the P2X 7 R cells is not externalized or activated by caspase 1  [ **CASP1** ] .  Ref: 11016935 J Biol Chem, 2001
- Further, elucidation of the mechanism of apoptosis induction in thymocytes with respect to the involvement of apoptosis related genes revealed that the death pathway followed an interleukin 1 beta  [ **IL1B** ]  converting enzyme  [ **CASP1** ]  dependent, Fas mediated apoptotic cascade, with a concomitant increase in the protein products of the bax, bad, p53, fas and fasL genes and cleavage of the 23 kD N terminal fragment of Bcl 2 that exhibited Bax like death effector properties.  Ref: 11006572 Tumour Biol< MedlineTA>Tumour Biol, 2001
- We therefore conclude that the interleukin 1 beta  [ **IL1B** ]  converting enzyme  [ **CASP1** ]  does not appear to be critical for cell death during the postnatal period but may favor motoneuron survival during adulthood.  Ref: 10869851 Neuroscience, 2000
- CONCLUSION ICE  [ **CASP1** ] , IL1 beta  [ **IL1B** ]  gene overexpression may be involved in the vulnerability of epithelial cell apoptosis in septic mice.  Ref: 10923469 Zhonghua Yi Xue Za Zhi, 1998
- The influences of interleukin 1 beta  [ **IL1B** ]  converting enzyme  [ **CASP1** ]  expression on the biologic characteristics of ovarian cancer cells < ArticleTitle>.  Ref: 11263197 Zhonghua Fu Chan Ke Za Zhi, 1999
- Similar results were obtained in interleukin 1 beta  [ **IL1B** ]  converting enzyme  [ **CASP1** ]  deficient and wild type mice, indicating that the interleukin 1 beta  [ **IL1B** ]  converting enzyme  [ **CASP1** ]  may not be required for caspase 3 activation.  Ref: 10869851 Neuroscience, 2000
- IL 1 beta  [ **IL1B** ]  converting enzyme  [ **CASP1** ]  caspase 1  [ **CASP1** ]  in intestinal inflammation.  Ref: 11606779 Proc Natl Acad Sci U S A, 2001
- In view of these findings, and in order to characterize the role of the interleukin 1beta converting enzyme in mediating or modulating cell death processes in vivo, we have investigated the effects of its deletion on motoneuron survival after a facial nerve transection in newborn and adult interleukin 1 beta  [ **IL1B** ]  converting enzyme  [ **CASP1** ]  knock out mice.  Ref: 10869851 Neuroscience, 2000
- The cellular Fas associated death domain like interleukin 1 beta  [ **IL1B** ]  converting enzyme  [ **CASP1** ]  inhibitory protein long form FLIP L, a structural homologue of caspase 8 lacking caspase activity because of several mutations in the active site, is a potent inhibitor of death receptor induced apoptosis.  Ref: 12215447 J Biol Chem, 2002
